# Untargeted Molecular Analysis of Exhaled Breath as a Diagnostic Test for Ventilator-Associated Lower Respiratory Tract Infections (BreathDx)

**DOI:** 10.1136/thoraxjnl-2021-217362

**Published:** 2021-06-04

**Authors:** Pouline MP van Oort, Tamara M Nijsen, Iain R White, Hugo H Knobel, Timothy Felton, Nicholas Rattray, Oluwasola Lawal, Murtaza Bulut, Waqar Ahmed, Antonio Artigas, Pedro R Povoa, Ignacio Martin-Loeches, Hans Weda, Royston Goodacre, Marcus J Schultz, Paul M Dark, Stephen J Fowler, Lieuwe D Bos, the BreathDx Consortium, Jonathan Bannard-Smith

**Affiliations:** 1 Intensive Care, Amsterdam UMC Location AMC, Amsterdam, The Netherlands; 2 Philips Research, Eindhoven, The Netherlands; 3 Laboratory for Environmental and Life Sciences, University of Nova Gorica, Nova Gorica, Slovenia; 4 Division of Infection, Immunity and Respiratory Medicine, University of Manchester, Manchester, UK; 5 Materials Analysis, Eurofins Materials Science Netherlands BV, Eindhoven, The Netherlands; 6 Department of Pharmacy, University of Strathclyde, Glasgow, UK; 7 Manchester Institute of Biotechnology, University of Manchester, Manchester, UK; 8 Critical Care Centre, Corporació Sanitària I Universitaria Parc Taulí-Hospital De Sabadell-Ciber Enfermedades Respiratorias, Sabadell, Barcelona, Spain; 9 Intensive Care, Centro Hospitalar de Lisboa Central, Lisbon, Portugal; 10 Department of Clinical Medicine, University of Dublin Trinity College, Dublin, Ireland; 11 Orikami, Nijmegen, The Netherlands; 12 Department of Biochemistry, University of Liverpool, Liverpool, UK; 13 Mahidol–Oxford Tropical Medicine Research Unit (MORU), Mahidol University, Bangkok, Thailand; 14 Intensive care, University of Manchester, Manchester, UK; 15 Manchester University NHS Foundation Trust – Wythenshawe Hospital, Manchester, UK; 16 Division of Infection, Immunity and Respiratory Medicine, NIHR’s Manchester Biomedical Research Centre (BRC), the University of Manchester, Manchester, UK; 17 Respiratory Medicine, Amsterdam UMC Location AMC, Amsterdam, Netherlands

**Keywords:** pneumonia, assisted ventilation, critical care

## Abstract

Patients suspected of ventilator-associated lower respiratory tract infections (VA-LRTIs) commonly receive broad-spectrum antimicrobial therapy unnecessarily. We tested whether exhaled breath analysis can discriminate between patients suspected of VA-LRTI with confirmed infection, from patients with negative cultures. Breath from 108 patients suspected of VA-LRTI was analysed by gas chromatography-mass spectrometry. The breath test had a sensitivity of 98% at a specificity of 49%, confirmed with a second analytical method. The breath test had a negative predictive value of 96% and excluded pneumonia in half of the patients with negative cultures. Trial registration number: UKCRN ID number 19086, registered May 2015.

## Introduction

Ventilator-associated lower respiratory tract infections (VA-LRTIs) are the most common nosocomial infections in the intensive care unit (ICU).^1^ Patients with suspected VA-LRTI usually receive broad-spectrum antibiotics before the diagnosis can be confirmed, since microbial cultures may take days to become positive.^2^ Volatile metabolites in exhaled breath can reflect changes in pathogen growth and/or the host response.^3^ Gas chromatography-mass spectrometry (GC-MS) is considered the gold standard for biomarker discovery from exhaled breath.^4^ Recent meta-analyses showed that the evidence is conflicting on the diagnostic value of volatile metabolites as biomarkers of VA-LRTI.^3 5^

In the current study, we hypothesised that exhaled breath analysis can discriminate between patients suspected of VA-LRTI and treated with broad-spectrum antibiotics who had positive cultures versus those who have negative cultures with high sensitivity and at least a moderate specificity.

## Methods

The ‘Molecular Analysis of Exhaled Breath as Diagnostic Test for Ventilator-Associated Pneumonia’ Study (BreathDx) was an international multicentre observational cohort study in ICU patients undergoing invasive ventilation and commencing antimicrobial therapy for suspected VA-LRTI. Patients were recruited across four ICUs of university hospitals in the Netherlands and the UK between February 2016 and February 2018. Since this study concerned patients lacking capacity, formal assent was sought with a designated consultee at time of inclusion. Deferred consent was obtained for patients who regained capacity. The study methods have been published.^6^

Patients were recruited and samples collected within 24 hours of the clinical suspicion of VA-LRTI. Exhaled breath samples were collected at first, followed by lower respiratory tract fluid samples (bronchoalveolar lavage (BAL) or mini-BAL samples). Positive cultures with a colony forming unit (CFU) cut-off of >10^4^ CFU/mL confirmed VA-LRTI. The specifications and origins of the equipment used for breath sampling have been described previously^6^ and met the criteria formulated in the European Respiratory Society technical statement on exhaled breath analysis.^7^ Breath metabolites were measured on two GC-MS machines with complementary properties. GC-MS-1 was targeted for more volatile metabolites, while GC-MS-2 targeted heavier and cyclic volatile metabolites. Data from GC-MS-1 were used for the primary analyses.

The sample size of 153 patients was not met in the chosen time frame for recruitment, due to an unexpected low presentation of VA-LRTI suspected cases at all study sites. Despite this, we maintained all predefined cut-offs for clinically relevant test characteristics.^6^ Untargeted analysis was used to investigate the primary outcome of the study. Sparse partial least squares (SPLS) models were used to fit diagnostic models on log-transformed data^8^ (for more details, see the online supplemental file 1).

10.1136/thoraxjnl-2021-217362.supp1 Supplementary data

## Results

**Figure 1:**
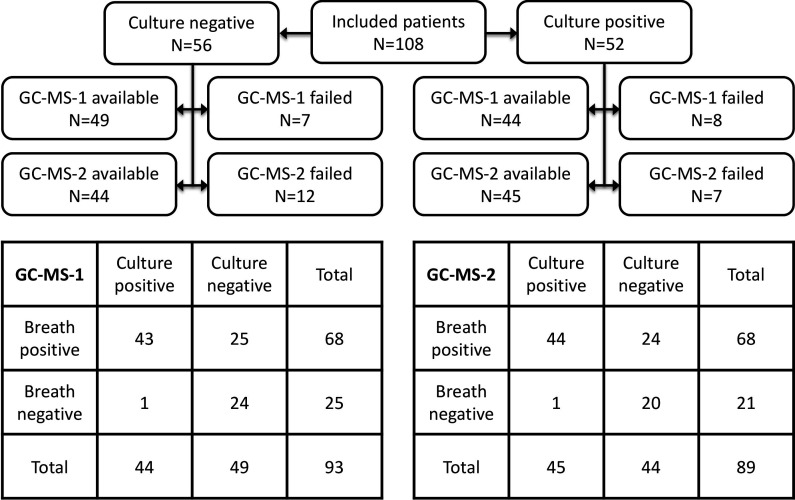
One hundred eight patients were included in the study. Exhaled breath analysis was performed using GC-MS-1 and GC-MS-2. Several measurements failed and were not used for further analysis. The 2×2 tables in the bottom of the figure indicate the diagnostic performance of each analytical platform for culture positivity. GC-MS, gas chromatography-mass spectrometry.

One hundred eight patients were suspected of VA-LRTI and were included, of whom 52 (48%) had positive cultures. Most patients developed VA-LRTI after 4 days of mechanical ventilation (68 of 108; 63%). Table 1 shows baseline demographic characteristics of the study population (stratified per centre; online supplemental table S1). Fifteen samples on GC-MS-1 and 19 on GC-MS-2 were of insufficient quality to use for further analysis (figure 1; baseline data stratified for availability of GC-MS-1 or 2 sample; online supplemental tables S2 and S3. Online supplemental figures S1–S9 show influence of centre, storage time, analysis date and duration of mechanical ventilation on breath profiles.

**Table 1:** Patient demographics

		Control (N=56)	VA-LRTI (N=52)
Age, years	Median (IQR)	59 (47–67)	59 (42–68)
Male	N (%)	41 (73.2)	31 (59.6)
Days on ICU*	Median (IQR)	9 (5–13)	7 (5–10)
Admission type	Medical—N (%)	32 (57.1)	18 (34.6)
	Emergency surgical—N (%)	15 (26.8)	16 (30.8)
	Planned surgical—N (%)	8 (14.3)	18 (34.6)
	Unscored—N (%)	1 (1.8)	0 0
Trauma	N (%)	13 (23.2)	20 (38.5)
Neurosurgery	N (%)	11 (19.6)	16 (30.8)
COPD	N (%)	6 (10.7)	8 (15.4)
ARDS	N (%)	4 (7.1)	0 (0)
CPIS	Median (IQR)	5 (4–6)	7 (5.8–7)
APACHE II	Median (IQR)	20 (15–23)	17 (11–22)
Temperature, °C	Median (IQR)	38 (37–39)	38 (37–38)
WCC, 10^9^/L	Median (IQR)	15 (10–21)	13 (12–18)
PaO_2_/FiO_2_, mm Hg	Median (IQR)	232 (156–270)	240 (171–284)
P_max_, cmH_2_O	Median (IQR)	20 (16–25)	21 (16–24)
PEEP, cmH_2_O	Median (IQR)	8 (5–10)	7.5 (5–10)
Tidal volume, mL	Median (IQR)	476 (417–550)	487 (411–602)
Confirmed VA-LRTI	VAP—N (%)		41 (79)
	VAT—N (%)		11 (21)
Culture results†	N (%)		
*Acinetobacter pittii*			1 (1.9)
*Enterobacter cloacae*			2 (3.8)
*Escherichia coli*			3 (5.8)
*Haemophilus influenzae*			5 (9.6)
*Klebsiella* spp			6 (11.5)
*Pseudomonas aeruginosa*			9 (17.3)
*Serratia marcescens*			2 (3.8)
*Staphylococcus aureus*‡			15 (28.8)
*Stenothrophomas maltophilia*			2 (3.8)
Other			7 (13.4)
ICU LOS, days	Median (IQR)	22 (14–33)	21 (15–32)
Hospital LOS, days	Median (IQR)	31 (15–44)	30 (19–57)
ICU mortality	N (%)	17 (30.4)	9 (17.3)

Continuous variables are expressed as median (25th–75th percentile); categorical variables as number of patients (percentage).

*Days on ICU until VA-LRTI suspicion.

†Potentially >1 cultured pathogen per patient.

‡All methicillin sensitive.

APACHE, Acute Physiology and Chronic Health Evaluation; ARDS, acute respiratory distress syndrome; CPIS, Clinical Pulmonary Infection Score; FiO_2_, inspired fraction of oxygen ratio; ICU, intensive care unit; LOS, length of stay; PEEP, positive end-expiratory pressure; P_max_, maximum airway pressure; VA-LRTI, ventilator-associated lower respiratory tract infection; VAP, ventilator-associated pneumonia; VAT, ventilator-associated tracheobronchitis; WCC, white cell count.

Five out of 184 unique volatile metabolites were significantly increased in patients suspected of VA-LRTI with positive cultures (table 2). SPLS analysis also identified these molecules, together with five additional volatile metabolites (table 2). The area under the receiver operating characteristics curve (AUROCC) for this model was 0.86 (95% CI: 0.79 to 0.94) with a specificity of 49% at the predefined sensitivity of 98% resulting in a negative predictive value (NPV) of 96% and a positive predictive value of 63% (figure 1). The addition of the clinical pulmonary infection score reduced the specificity to 41% at the same sensitivity with a marginal increase in AUROCC to 0.87 (95% CI: 0.80 to 0.94). This accuracy was reproduced in additional samples collected at the same time and analysed on the same platform and on GC-MS-2 (online supplemental table S4 and figure S11). No confounding variables were identified (online supplemental table S5). The association between the breath test and confirmed VA-LRTI was not moderated by the presence of pulmonary infiltrates (p=0.17) or if it concerned early or late VA-LRTI (p=0.40).

**Table 2:** VOCs included in the diagnostic model for GC-MS-1 for culture positivity

VOC ID	Suspected origin	MSI level	Abundance	Loadings	
Formaldehyde	Endogenous	2	↑*	−0.33	0.14
Tetrahydrofuran	Unknown	2	↑	−0.28	0.41
3-methylheptane	Endogenous	2	↓	0.05	−0.69
Branched alkane	Unknown	3	↑*	−0.38	−0.10
Dimethylsulfide	Endogenous	2	↑*	−0.33	0.31
6-methyl-5-hepten-2-one	Endogenous	2	↑	−0.31	−0.22
Branched alkane	Unknown	2	↑	−0.31	−0.35
2,2,4,4-tetramethyloctane	Unknown	2	↑*	−0.34	−0.20
Enflurane	Exogenous	2	↑	−0.31	0.08
2,2-dimethyldecane	Endogenous	2	↑*	−0.39	−0.10

Abundance of the compound was either increased (↑) or decreased (↓) in the breath of patients with positive cultures. Loadings show the loading factors to the two projected components in the SPLS-DA model.

*Also significant in univariate modelling shown in Volcano plot. Endogenous indicates that a molecule likely originates from host or from bacteria. Exogenous indicates that a molecule is likely to come from the environment and thus is a false discovery. Unknown indicates that no clear link with either pathophysiological process is known.

GC-MS, gas chromatography-mass spectrometry; ID, identity; MSI, Metabolomics Standards Initiative; SPLS-DA, sparse partial least squares-discriminant analysis; VOCs, volatile organic compounds.

## Discussion

In the present study, 53% of the included patients had negative cultures and may have received antibiotics unnecessarily. Exhaled breath analysis correctly suggested the absence of a bacterial growth in half of these patients. A high sensitivity accompanied by an acceptable specificity is required to allow for withholding of antibiotics for VA-LRTI. The presented results need to be further triangulated with additional biomarker data and comparison of composition of the lung microbiome. Compared with previous studies that focused on breath analysis, this is the first to predefine these diagnostic cut-offs in an analysis plan and commit to a standardised analysis methodology.

A variety of volatile organic compounds contributed to the diagnostic model purposed to exclude respiratory infection. Ten of the 21 molecules have been described previously in relation to bacterial growth or host response, increasing the biological plausibility of our findings. Formaldehyde and dimethylsulfide were predictive biomarkers for respiratory infection based on a systematic review of preclinical data.^9^ Additionally, several of the identified hydrocarbons have been linked to oxidative stress. For example, 3-methylheptane has been associated with acute respiratory distress syndrome,^10 11^ is associated with lipid peroxidation^12^ and has also been detected in the bacterial headspace samples of *Escherichia coli.* 12

The major strength of this study is that we followed a predefined methodology and statistical analysis plan as published in the study protocol.^6^ Another strength was the multicentre design of the study, enhancing the chances of reaching the required sample size, sampling patients in a broader range of clinical settings and promoting the subsequent generalisation of the study results. The BreathDx Study faced a slower recruitment rate than expected, which is a weakness of the study. The calculated required sample size of 153 patients was not reached. An unexpected low incidence of VA-LRTI suspicion was seen at all sites. A larger sample size would have resulted in more confidence in the estimated sensitivities and specificities. An independent validation of the found accuracies is required, preferably using a bedside technology that can be used in clinical practice. It would be preferable to collect samples before antibiotic administration rather than within 24 hours as we did in this study. Incorporation with other clinical and biological markers is encouraged and should be part of future studies.

To conclude, exhaled breath analysis can differentiate between patients with confirmed VA-LRTI and those with negative cultures with high NPV. The identified biomarkers are supported by preclinical evidence.
