# Supplementary material for: Untargeted Molecular Analysis of Exhaled Breath as a Diagnostic Test for Ventilator-Associated Lower Respiratory Tract Infections (BreathDx)
Source: Thorax. 2021 Jun 4;77(1):79–81. doi: 10.1136/thoraxjnl-2021-217362 (PMC8685633; doi:10.1136/thoraxjnl-2021-217362)
Supplement: Supplementary data [file thoraxjnl-2021-217362supp001.pdf]

Online supplementary material

Molecular Analysis of Exhaled Breath as a Diagnostic Test for Ventilator–Associated Lower Respiratory Tract Infections (BreathDx) – an international multicentre observational study

Pouline M. P. van Oort[1], Tamara Nijsen[2], Iain White[3], Hugo Knobel[4], Timothy Felton[5,6], Nicholas J. W. Rattray[7], Oluwasola Lawal[6], Murtaza Bulut[2], Waqar M. Ahmed[3], Antonio Artigas[8], Pedro Póvoa[9], Ignacio Martin-Loeches[10], Hans Weda[11], Royston Goodacre[3,12], Marcus J. Schultz[1,13], Paul Dark[5,6], Stephen J. Fowler[5,6†] and Lieuwe D. J. Bos[1†*] on behalf of the BreathDx Consortium[+]

*Correspondence: l.d.bos@amsterdamumc.nl, tel. +31613901359

[1]Department of Intensive Care, Amsterdam UMC – location Academic Medical Centre (AMC), Meibergdreef 9, 1105 AZ, Amsterdam, the Netherlands.

[2]Philips Research, High Tech Campus 34, 5656 AE, Eindhoven, the Netherlands.

[3]Manchester Institute of Biotechnology (MIB), the University of Manchester, 131 Princess Street, Manchester, M1 7DN, UK.

[4]Eurofins Materials Science Netherlands BV, High Tech Campus 11, 5656 AE, Eindhoven, the Netherlands

[5]Manchester University NHS Foundation Trust – Wythenshawe Hospital, Southmoor Rd, Wythenshawe, Manchester, M23 9LT, UK.

[6]Division of Infection, Immunity and Respiratory Medicine, NIHR's Manchester Biomedical Research Centre (BRC), the University of Manchester, 29 Grafton St, Manchester, M13 9WU, UK.

[7]Department of Pharmacy, University of Strathclyde, 16 Richmond St, Glasgow, G1 1XQ, UK.

van Oort PMP, *et al. Thorax* 2021; 77:79–81. doi: 10.1136/thoraxjnl-2021-217362

[8]Critical Care Department, CIBER Enfermedades Respiratorias, Corporacion Sanitaria Universitaria Parc Tauli, Parc Tauli 1, 08208 Sabadell, Barcelona, Spain.

[9]Hospital de São Francisco Xavier, Centro Hospitalar Lisboa Ocidental, NOVA Medical School, New University of Lisbon, Estr. Forte do Alto Duque, 1449-005, Lisbon, Portugal.

[10]Department of Clinical Medicine, St James's Hospital, Multidisciplinary Intensive Care Research Organization (MICRO), Trinity Centre for Health Sciences, Dublin 8, James' St, Ireland.

[11]Orikami, Ridderstraat 29, 6511 TM, Nijmegen, the Netherlands

[12]Department of Biochemistry, University of Liverpool, Institute of Integrative Biology, Liverpool, L69 3BX, UK.

[13]Mahidol–Oxford Tropical Medicine Research Unit (MORU), Mahidol University, 420/6 Rajvithi Road, Tungphyathai, Bangkok 10400, Thailand

[†]Authors contributed equally.

Available ORCID ID's:
L.D. Bos (corresponding author): 0000-0003-2911-4549
P.M.P. van Oort: 0000-0002-2719-8817
H. Knobel: 0000-0002-3548-4928
N. Rattray: 0000-0002-3528-6905
O. Lawal: 0000-0002-7134-4930
W. Ahmed: 0000-0003-1490-6391
A. Artigas: 0000-0002-8029-1017
P. Povoa: 0000-0002-7069-7304
I. Martin-Loeches: 0000-0002-5834-4063
H. Weda: 0000-0001-6675-2270
R. Goodacre: 0000-0003-2230-645X
P. Dark: 0000-0003-3309-0164
S. Fowler: 0000-0002-4524-1663

Online methods

Design and ethical considerations

The ‘Molecular Analysis of Exhaled Breath as Diagnostic Test for Ventilator–Associated Pneumonia’ – study (BreathDx) was an international multicentre observational cohort study in ICU patients undergoing invasive ventilation and commencing antimicrobial therapy for suspected VA-LRTI. Patients were recruited across four ICUs of university hospitals in the Netherlands and the United Kingdom: Amsterdam UMC – location AMC in Amsterdam, the Netherlands; Manchester University NHS Foundation Trust – Wythenshawe Hospital (WH), Manchester University NHS Foundation Trust – Manchester Royal Infirmary (MRI) and Salford Royal NHS Foundation Trust (SRFT) in Manchester, United Kingdom. Patients were recruited over a 24-month period, between February 2016 and February 2018. The study was approved by the institutional review boards of the individual institutions and, in the case of the UK, by a National Health Service Research Ethics Committee. Since this study concerned patients lacking capacity, formal assent was sought with a designated consultee at time of inclusion. Deferred consent was obtained for patients who regained capacity. BreathDx was registered at UK Clinical Research Network (ID number 19086). The study methods have been published [1].

Patients

Inclusion criteria were: (1) age 18 years or older; (2) intubation and mechanical ventilation for 48 hours or more; and (3) clinical suspicion of VA-LRTI by the treating physician followed by the initiation of broad-spectrum antibiotics. Patients who were deemed inappropriate to collect samples from (e.g. patients receiving end-of-life care) were excluded. Patients in strict isolation (e.g. in case of Middle East Respiratory Syndrome, Ebola or resistant tuberculosis) were also excluded.

Standard care

In all hospitals heat and moisture exchangers (HMEs) were used and there was focussed training on hand hygiene control measures.

As part of standard care for prevention of nosocomial infections, patients at the ICU of the AMC in Amsterdam received selective decontamination of the digestive tract (SDD). The antibiotic protocol for suspected VA-LRTI was intravenous treatment with amoxicillin/clavulanic acid and ceftazidime at this site. No SDD was used on the ICUs of the three Manchester based hospitals. For Wythenshawe hospital the antibiotic treatment for VA-LRTI consisted of amoxicillin/clavulanic acid when VA-LRTI developed before day 5 of mechanical ventilation while piperacillin-tazobactam was started after day 5. On the ICU of Salford Royal, amoxicillin/clavulanic acid was used as first line treatment for VA-LRTI in case of less than 7 days of hospitalization; at day 7 or beyond piperacillin-tazobactam was administered. On Manchester Royal Infirmary's ICU the first choice was piperacillin-tazobactam (or meropenem in case of known colonisation with an Extended Spectrum Beta-Lactamase (ESBL)), aimed for de-escalation to amoxicillin/clavulanic acid as soon as cultures and sensitivities were confirmed.

Sample size calculation and study endpoints

The sample size calculation has been described before [1]. The intended use of our breath test is to refute a diagnosis of a respiratory infection and to withhold antibiotics in patients clinically suspected of VA-LRTI. The primary reference test was culture positive / negative VA-LRTI. For this purpose, a test with a very high sensitivity is required to minimise false-negative results. Predefining a sensitivity of 98-100% and with a lower 95% confidence-boundary of 90% sensitivity, resulted in 61 estimated required cases with culture positivity [2]. We assumed a prevalence of 40% of positive cultures of BAL in suspected VA-LRTI patients based on literature [3]. It was estimated that a total study sample

van Oort PMP, *et al. Thorax* 2021; 77:79–81. doi: 10.1136/thoraxjnl-2021-217362

size was 153 subjects was required to meet these criteria. Taken together with high sensitivity this would result in 95-100% NPV, theoretically sufficient to withhold antibiotics.

Definitions of ventilator-associated lower respiratory tract infections

Suspected VAP was defined by: (1) clinical suspicion for VA-LRTI by the physician based on signs of infection [temperature >38 or <36.5°C; white blood cell count <4,000 or >12,000/mm^3, purulent tracheal secretions], and (2) new infiltrates on chest X-ray. Suspected VAT did not require the second criterion. Positive (mini)-BAL cultures with a colony forming unit (CFU) cut-off of >10^4 CFU/mL confirmed VA-LRTI and this was used as the reference test.

Study procedures

Patients were recruited and samples were collected within 24 hours of the clinical suspicion of VAP. Exhaled breath samples were collected at first, followed by lower respiratory tract fluid samples (BAL or mini-BAL samples). Breath samples were sent for analysis within two days of collection and analysed within two weeks of arrival. The (mini-)BAL samples were processed and frozen immediately at -80°C after recruitment. Clinical information, reference standard results and index test results were available to the researchers without blinding.

Bronchoalveolar lavage

A broncho-alveolar lavage (BAL) sample was obtained for microbiological analysis directly after collection of the breath samples by bronchoscopy or through a non-directed appraoch. Bronchoscopic BAL was performed according to the BTS guidelines [4]. For non-directed BAL, a syringe was connected to a 50cm suctioning catheter, followed by an injection of 20mL 0.9% saline

van Oort PMP, *et al. Thorax* 2021; 77:79–81. doi: 10.1136/thoraxjnl-2021-217362

BMJ Publishing Group Limited (BMJ) disclaims all liability and responsibility arising from any reliance placed on this supplemental material which has been supplied by the author(s)

into the airway. At least 4mL was aspirated of which 1mL was used for culturing. A semi-quantitative bacterial count with a cut-off of 10^4 CFU/mL defined a positive culture.

Breath sampling and analysis

The specifications and origins of the equipment used for breath sampling have been described previously [1], and met the criteria formulated in the ERS technical statement on exhaled breath analysis [5]. Breath samples were collected at one time point using a breath gas sampler (BGS) containing a pump and a mass flow controller. This BGS was used together with PTFE (Polytetrafluoroethylene) tubing. 1200mL of exhaled breath was collected onto stainless steel sorbent tubes using the method described in the protocol [1]. All samples were collected in duplicate and link-anonymised. Subsequently, the samples were sent to two different laboratory locations for analysis (one pair to Philips Research, Eindhoven, the Netherlands, and the other to the Manchester Institute of Biotechnology, University of Manchester, UK). The samples that were collected for analysis at Philips Research, the exhaled breath was collected using sorbent tubes packed with 300mg Carbograph 5TD (Markes International, Llantrisant, UK) and 90mg Tenax GR (Sigma-Aldrich Chemie B.V., Zwijndrecht, the Netherlands). The samples that were destined to be analysed at the Manchester Institute of Biotechnology were collected using 200mg Tenax GR (Markes International, Llantrisant, UK) sorbent tubes. The duplicate sample was used to assess repeatability of the measurement. Importantly, the machines were designed to capture a different fraction of exhaled metabolites and were not set-up to replicate, but rather to provide a comprehensive overview of the VOCs in the breath of patients with suspected VA-LRTI.

Statistical analysis

The sample size was not met in the chosen time frame for recruitment, due to an unexpected low presentation of VA-LRTI suspected cases at all study sites. Despite this, we maintained all predefined cut-offs for clinically relevant test characteristics. Data were visually inspected for exclusion of contaminated samples, failed chromatography runs, and technical errors prior to any link to the clinical characteristics of the patient. Unreliable or failed breath measurements were not included in the subsequent analysis (figure 1). The method for peak detection and alignment has been explained in the BreathDx protocol paper [1]. The resulting peak table was used for data exploration and untargeted analysis. Data exploration was performed using principal component analysis (PCA) on $\log_{10}$-transformed and scaled data. Major potential influential factors such as the replacement of GC columns and other instrument parts, recruitment centres, duration of sample storage, and duration of mechanical ventilation were assessed by data visualisation.

Each row in the subsequent ion-fragment peak table corresponded with a sample. Columns contained sample and patient metadata (e.g. sample data and clinical characteristics), and the abundances of the peaks or ion-fragments at a particular retention time: a few thousand depending on the analytical platform. This table served as input for the following statistical analysis. Volatile organic compounds (VOCs; referred to in the text as volatile metabolites) were identified using the National Institute of Standards and Technology library on both GC-MS platforms [6] and the certainty of identification was reported with Metabolomics Standard Initiative (MSI) levels [7].

Untargeted analysis was used to investigate the primary outcome of the study. First, VOCs were studied individually using multiple linear models by generalized least squares with the *limma* R-package, resulting in adjusted *p*-values and fold changes. These were visualised in Volcano plots. For multi-dimensional data analysis, sparse partial least squares (SPLS) models were fitted on the log-transformed data, as described previously [8]. VOCs were chosen if the algorithm selected the VOC in more than 40% of permutated scenarios indicating stability of selection. The data could not be split into a training set and a validation set due to the relatively small number of patients. Instead,

permutation tests were used to evaluate the performance of the model and the correct classification rate (CCR) based on random label permutation was reported.

The moderation of effect by the presence of: 1) new infiltrates (VAP *versus* VAT); 2) the early *versus* late development of VA-LRTI was assessed by including these factors as an interaction term in a logistic regression model. The influence of potential confounders (e.g. comorbidities, ventilator settings, medications) on the association between exhaled breath and VA-LRTI was investigated. For this the log odds ratios were compared between a logistic regression model with the VOCs of interest as dependent variables and VA-LRTI (yes/no) as independent variable, and the same model with the inclusion of the potential confounder as co-variate. The co-variate was considered a confounder when a change of ≥ 10% was observed for the log odds ratio. The odds ratios with confidence intervals are presented for the association between the breath test and proven VAP and for models that include all potential confounders as co-variate.

van Oort PMP, *et al*. *Thorax* 2021; 77:79–81. doi: 10.1136/thoraxjnl-2021-217362

Online Tables

Table S1. Patient characteristics per study site

		AMC (*N*= 57)		MRI (*N*= 17)		Salford (*N*= 31)		WH (*N*= 3)	
**Age *(years)***	*Median (IQR)*	60	(49.8-68)	71	(64-78)	48.5	(29.2-58.8)	51	(48.5-57.5)
**Male**	*N (%)*	42	(73.7)	9	(52.9)	20	(64.5)	1	(33.3)
**Days on ICU***	*Median (IQR)*	9	(6-13)	7	(3.5-13)	6	(4-9)	12	(7.5-13)
**Admission type**	*Medical – N (%)*	33	(57.9)	9	(52.9)	5	(16.1)	3	(100.0)
	Emergency surgical – N (%)	14	(24.6)	2	(11.8)	15	(48.4)	0	0
	Planned surgical – N (%)	10	(17.5)	5	(29.4)	11	(35.5)	0	0
	Unscored – N (%)	0	0	1	(5.9)	0	0	0	0
**Trauma**	*N (%)*	10	(17.5)	2	(11.8)	20	(64.5)	1	(33.3)
**Neurosurgery**	*N (%)*	7	(12.3)	2	(11.8)	18	(58.1)	0	0
**COPD**	*N (%)*	10	(17.5)	3	(17.6)	0	0	1	(33.3)
**ARDS**	*N (%)*	4	(7.0)	0	0	0	0	0	0
**CPIS**	*Median (IQR)*	5	(4-6)	6	(4-6)	7	(6-7)	5	(3.5-6)
**APACHE II**	*Median (IQR)*	20	(15-24)	17	(14-25)	11	(8.5-17.5)	15	(9-18.5)
**Temperature *(°C)***	*Median (IQR)*	38	(37-39)	37	(36-38)	38	(38-39)	37	(36.5-37)
**WCC *(10^9/L)***	*Median (IQR)*	15.5	(12-21.2)	13	(8.8-17.2)	12	(10.2-14.8)	19	(13-21.5)
**PaO_2/FiO_2 *(mmHg/%)***	*Median (IQR)*	195	(157.5-267)	232.5	(183.8-288.8)	240	(198.8-315)	262.5	(210-281.2)
**P_{max} *(cmH_2O)***	*Median (IQR)*	20	(15-27.5)	20	(17.8-22)	21	(17-24)	20	(15-24)
**PEEP *(cmH_2O)***	*Median (IQR)*	7	(5-10)	10	(8-10)	7	(5-8)	5	(5-8.5)
**Tidal volume *(mL)***	*Median (IQR)*	452	(370-536)	475.5	(455.2-572)	565	(483-614)	450	(408-588)
**No VA-LRTI**	*N (%)*	38	(66.7)	8	(47.1)	8	(25.8)	2	(66.7)
**VA-LRTI**	*N (%)*	19	(33.3)	9	(52.9)	23	(74.2)	1	(33.3)
**Culture results****	*N (%)*								

van Oort PMP, *et al. Thorax* 2021; 77:79–81. doi: 10.1136/thoraxjnl-2021-217362

Acinetobacter pittii		0	0	0	0	1	(3.2)	0	0
Enterobacter cloacae		2	(3.5)	0	0	0	0	0	0
Escherichia coli		0	0	0	0	2	(6.5)	1	(33.3)
Haemophilus influenzae		0	0	0	0	5	(16.1)	0	0
Klebsiella spp.		1	(1.8)	3	(17.7)	2	(6.5)	0	0
Pseudomonas aeruginosa		6	(10.5)	1	(5.9)	2	(6.5)	0	0
Serratia marcescens		1	(1.8)	1	(5.9)	0	0	0	0
Staphylococcus aureus		4	(7.0)	2	(11.8)	9	(29.0)	0	0
Stenothrophomas maltophilia		2	(3.5)	0	0	0	0	0	0
**ICU LOS *(days)***	*Median (IQR)*	22	(14-32)	21.5	(11-37.2)	21	(15-28)	33	(19.5-34)
**Hospital LOS *(days)***	*Median (IQR)*	29	(15-44)	33	(21.2-64.5)	22	(18-45)	45.5	(44.8-46.2)
**ICU mortality**	*N (%)*	20	(35.1)	2	(11.8)	3	(9.7)	1	(33.3)

Table S1. Patient characteristics per study site. Continuous variables are expressed as median (25th–75th percentile); categorical variables as number of patients (percentage). *days on ICU until VA-LRTI suspicion. ** Potentially >1 cultured pathogen per patient. IQR = interquartile range; AMC = Academic Medical Centre, Amsterdam; MRI = Manchester Royal Infirmary; WH = Wythenshawe hospital; ICU = intensive care unit; COPD = chronic obstructive pulmonary disease; ARDS = acute respiratory distress syndrome; CPIS = clinical pulmonary infection score; APACHE = Acute Physiology and Chronic Health Evaluation; WCC = white cell count; PaO_2/FiO_2 = partial pressure of oxygen / inspired fraction of oxygen ratio; P_{max} = maximum airway pressure; PEEP = positive end-expiratory pressure; LOS = length-of-stay.

Table S2: Patient demographics for patients with GC-MS-1 sample available

		Control (*N*=49)		VA-LRTI (*N*=44)	
**Age, *years***	*Median (IQR)*	59	(46-67)	59	(42.5-69)
**Male**	*N (%)*	37	(75.5)	27	(61.4)
**Days on ICU***	*Median (IQR)*	9	(5.8-13.5)	6	(4-9)
**Admission type**	*Medical – N (%)*	30	(61.2)	15	(34.1)
	Emergency surgical – N (%)	12	(24.5)	15	(34.1)
	Planned surgical – N (%)	6	(12.2)	14	(31.8)
	Unscored – N (%)	1	(2)	0	0
**Trauma**	*N (%)*	6	(12.2)	14	(31.8)
**Neurosurgery**	*N (%)*	9	(18.4)	15	(34.1)
**COPD**	*N (%)*	5	(10.2)	8	(18.2)
**ARDS**	*N (%)*	4	(8.2)	0	(0)
**CPIS**	*Median (IQR)*	5	(4-6)	7	(5.8-7)
**APACHE II**	*Median (IQR)*	20	(15-22)	16	(10-21.2)
**Temperature, *°C***	*Median (IQR)*	38	(37-39)	38	(37-38)
**WCC, *10^9/L***	*Median (IQR)*	15	(10.5-21.5)	13	(11.5-17.5)
**PaO_2/FiO_2, *mmHg***	*Median (IQR)*	231	(157.5-268.5)	240	(172.5-283.5)
**P_{max}, *cmH_2O***	*Median (IQR)*	20	(15.8-25.5)	20	(16-24)
**PEEP, *cmH_2O***	*Median (IQR)*	8	(5-10)	7.5	(5-10)
**Tidal volume, *mL***	*Median (IQR)*	8	(5-10)	7,5	(5-10)
**Confirmed VA-LRTI**	*VAP – N (%)*			36	(81.8)
	VAT – N (%)			8	(18.2)
**Culture results****	*N (%)*				
Acinetobacter pittii				1	(2.3)
Enterobacter cloacae				1	(2.3)
Escherichia coli				2	(4.5)
Haemophilus influenzae				5	(11.4)
Klebsiella spp.				6	(13.6)
Pseudomonas aeruginosa				9	(20.5)
Serratia marcescens				1	(2.3)
Staphylococcus aureus**				12	(27.3)
Stenothrophomas maltophilia				2	(4.5)
Other				5	(11.4)
**ICU LOS, *days***	*Median (IQR)*	20	(14-32.2)	21	(15.2-27.8)
**Hospital LOS, *days***	*Median (IQR)*	27	(14.5-41.5)	28	(17-44)
**ICU mortality**	*N (%)*	16	(32.7)	8	(18.2)

Table S2. Patient demographics for patients with sample available on GC-MS-1. Continuous variables are expressed as median (25th–75th percentile); categorical variables as number of patients (percentage). *days on ICU until VA-LRTI suspicion. **Potentially >1 cultured pathogen per patient. ****All methicillin sensitive. IQR = interquartile range; ICU = intensive care unit; COPD = chronic obstructive pulmonary disease; ARDS = acute respiratory distress syndrome; CPIS = clinical pulmonary infection score; APACHE = Acute Physiology and Chronic Health Evaluation; WCC = white cell count; PaO_2/FiO_2 = partial pressure of oxygen / inspired fraction of oxygen ratio; P_{max} = maximum

airway pressure; PEEP = positive end-expiratory pressure; VA-LRTI = Ventilator associated lower respiratory tract infection; VAP = Ventilator associated pneumonia; VAT = Ventilator associated tracheobronchitis; LOS = length-of-stay.

Table S4: Patient demographics for patients with GC-MS-2 sample available

		**Control (*N*=44)**		**VA-LRTI (*N*=45)**	
**Age*, years***	*Median (IQR)*	59	(47.5-66)	58,5	(39.8-68.2)
**Male**	*N (%)*	30	(68.2)	27	(60)
**Days on ICU***	*Median (IQR)*	8	(4-13.5)	6	(4-9.5)
**Admission type**	*Medical – N (%)*	27	(61.4)	15	(33.3)
	Emergency surgical – N (%)	10	(22.7)	14	(31.1)
	Planned surgical – N (%)	6	(13.6)	16	(35.6)
	Unscored – N (%)	1	(2)	0	0
**Trauma**	*N (%)*	9	(20.5)	18	(40)
**Neurosurgery**	*N (%)*	9	(20.5)	18	-40
**COPD**	*N (%)*	5	(11.4)	6	(13.3)
**ARDS**	*N (%)*	1	(2.3)	0	0
**CPIS**	*Median (IQR)*	5	(4-6)	7	(6-7)
**APACHE II**	*Median (IQR)*	20	(14.8-23.2)	17	(10-22)
**Temperature, *°C***	*Median (IQR)*	38	(37-39)	38	(37-38)
**WCC, *10^9/L***	*Median (IQR)*	14	(9.2-17)	13	(12-18)
**PaO_2/FiO_2*, mmHg***	*Median (IQR)*	236	(161-285)	246	(178-285)
**P_{max}*, cmH_2O***	*Median (IQR)*	20	(16-25)	21	(16-25)
**PEEP, *cmH_2O***	*Median (IQR)*	8	(5-10)	7	(5-10)
**Tidal volume*, mL***	*Median (IQR)*	479	(417-566)	468	(390-588)
**Confirmed VA-LRTI**	*VAP – N (%)*			34	(75.6)
	VAT – N (%)			11	(24.4)
**Culture results****	*N (%)*				
Acinetobacter pittii				1	(2.2)
Enterobacter cloacae				2	(4.4)
Escherichia coli				2	(4.4)
Haemophilus influenzae				5	(11.1)
Klebsiella spp.				5	(11.1)
Pseudomonas aeruginosa				8	(17.8)
Serratia marcescens				2	(4.4)
Staphylococcus aureus**				13	(28.9)
Stenothrophomas maltophilia				1	(2.2)
Other				7	(15.6)
**ICU LOS*, days***	*Median (IQR)*	21	(13.5-33)	21	(15-30.5)
**Hospital LOS*, days***	*Median (IQR)*	31	(14.8-45.5)	30	(19.5-50)
**ICU mortality**	*N (%)*	11	(25)	7	(15.6)

Table S3. Patient demographics for patients with sample available on GC-MS-2. Continuous variables are expressed as median (25th–75th percentile); categorical variables as number of patients (percentage). *days on ICU until VA-LRTI suspicion. **Potentially >1 cultured pathogen per patient. ****All methicillin sensitive. IQR = interquartile range; ICU = intensive care unit; COPD = chronic obstructive pulmonary disease; ARDS = acute respiratory distress syndrome; CPIS = clinical pulmonary infection score; APACHE = Acute Physiology and Chronic Health Evaluation; WCC = white cell count; PaO_2/FiO_2 = partial pressure of oxygen / inspired fraction of oxygen ratio; P_{max} = maximum

airway pressure; PEEP = positive end-expiratory pressure; VA-LRTI = Ventilator associated lower respiratory tract infection; VAP = Ventilator associated pneumonia; VAT = Ventilator associated tracheobronchitis; LOS = length-of-stay.

Table S4: VOCs included in the diagnostic model for GC-MS-2 for culture positivity.

VOC ID	Suspected origin	MSI level	Abundance	Loadings	
**isopropylbenzene**	Endogenous	2	↓	-0.29	-0.34
**2-propenylbenzene**	Unknown	2	↓	-0.34	-0.27
**1-propenylbenzene**	Unknown	2	↓	-0.34	-0.23
**2,6-difluorobenzaldehyde**	Exogenous	2	↑	0.26	-0.48
**2-bromophenol**	Exogenous	2	↓	-0.26	0.45
**m-di-tert-butylbenzene**	Unknown	2	↓	-0.32	-0.21
**cyclohexane**	Endogenous	2	↓	-0.33	-0.06
**2,6,7-trimethyldecane**	Unknown	2	↑	0.34	-0.04
**2-methyldecane**	Endogenous	2	↑	0.31	-0.10
**3-methylheptane**	Endogenous	2	↓	-0.33	-0.01
**cyclohexanol**	Endogenous	2	↑	0.13	-0.51

Table S4. VOCs included in the diagnostic model for GC-MS-2 for prediction of positive cultures. Abundance of the compound was either increased (↑) or decreased (↓) in the breath of patients with positive cultures. Loadings show the loading factors to the two projected components in the sPLS-DA model.;VOC = volatile organic compound; IC = identity; MSI = Metabolomics Standards Initiative. *Also significant in univariate modelling shown in Volcano plot. Endogenous indicates that a molecule likely originates from host or from bacteria. Exogenous indicates that a molecule is likely to come from the environment en thus is a false-discovery. Unknown indicates that no clear link with either is known.

van Oort PMP, *et al. Thorax* 2021; 77:79–81. doi: 10.1136/thoraxjnl-2021-217362

Table S5. Potential confounders for diagnostic accuracy of breath test based on GC-MS-1

**Breath test (Base model)**	**OR**	**OR lower limit CI**	**OR higher limit CI**	
**Breath test**	11.4	4.2	31.5	
**Addition of potential confounder**	**Adjusted OR**	**Adjusted OR lower limit CI**	**Adjusted OR upper limit CI**	**Change in β coefficient of likelihood of infection based on breath test**
**+ Duration of MV before VA-LRTI**	10.2	3.6	28.4	5.9%
**+ Infection at admission**	9.9	3.5	27.8	5.2%
**+ COPD**	13.1	4.5	28.3	5.8%
**+ P_{max}**	11.8	4.2	32.8	0.2%
**+ PEEP**	11.1	4.0	30.4	1.9%
**+ FiO_2**	12.3	4.4	34.7	3.2%
**+ $etCO_2$**	11.7	3.9	34.9	3.0%

Odds ratio with confidence interval for the breath test and confirmed VA-LRTI and the influence of potential confounders on this relationship. MV = mechanical ventilation; VA-LRTI = ventilator-associated-lower respiratory tract infection; COPD = chronic obstructive pulmonary disease; P_{max} = maximum airway pressure; PEEP = positive end-expiratory pressure; FiO_2 = inspired fraction of oxygen; $etCO_2$ = end-tidal CO_2.

van Oort PMP, *et al. Thorax* 2021; 77:79–81. doi: 10.1136/thoraxjnl-2021-217362

Online figures

Figure S1: Influence of centre on principal components derived from GC-MS-1

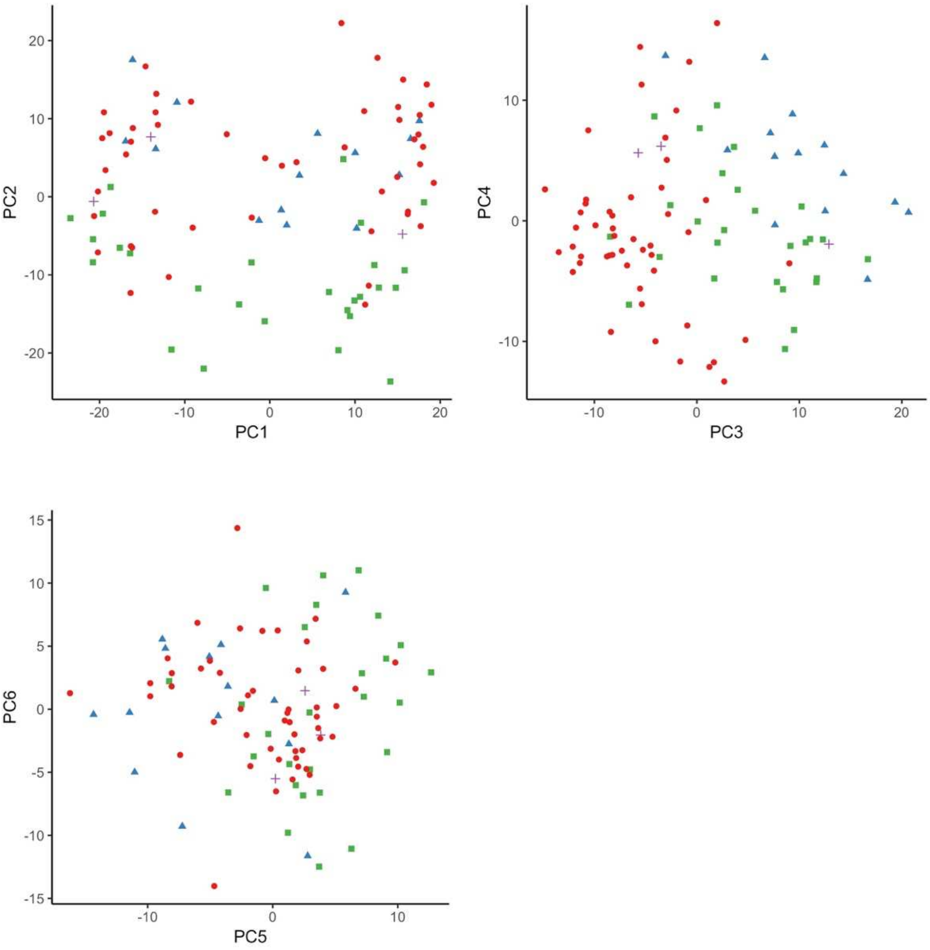

Centres: red dot (●): AMC Amsterdam; blue triangle (▲): Manchester Royal Infirmary; green square (■): Salford; purple cross (+): Wythenshawe hospital .

Interpretation: The centres are not well differentiated on the first two PCs but can be discriminated based on PC3. A small portion of the variation in exhaled metabolites is thus explained by the centre the patient is recruited.

van Oort PMP, *et al. Thorax* 2021; 77:79–81. doi: 10.1136/thoraxjnl-2021-217362

Figure S2: Influence of analysis date on principal components derived from GC-MS-1

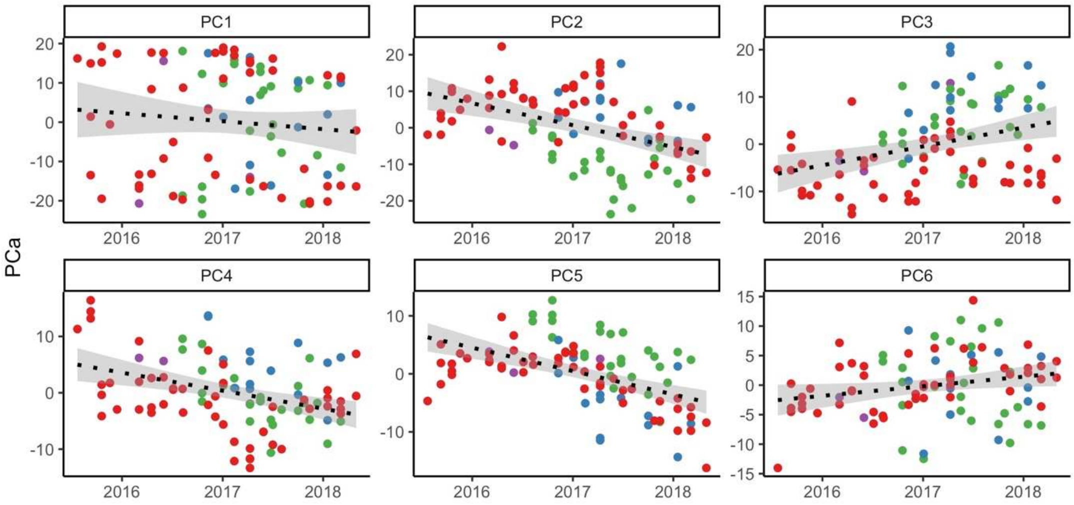

Figure S2. The first six PCs that explain the highest amount of variance are depicted. Dots represent the collected breath samples from either AMC Amsterdam (red ●), Manchester Royal Infirmary (MRI) (blue ●), Salford Trust (green ●), and Wythenshawe Hospital (purple ●). Dashed line = linear regression line; shaded areas = 95% confidence interval of linear regression line.

Interpretation: There are no strong changes in the PCs over time, except for PC3, but this is explained by the addition of other centres as shown in Figure S1.

van Oort PMP, *et al. Thorax* 2021; 77:79–81. doi: 10.1136/thoraxjnl-2021-217362

Figure S3: Influence of storage time on principal components derived from GC-MS-1

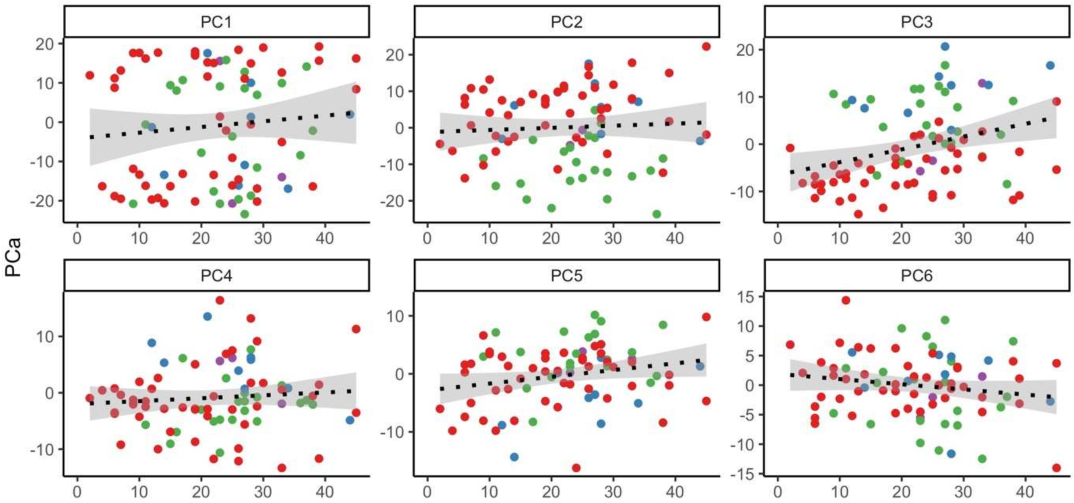

Figure S3. Dots represent the collected breath samples either from AMC Amsterdam (red ●), Manchester Royal Infirmary (MRI) (blue ●), Salford Trust (green ●), and Wythenshawe Hospital (purple ●). Dashed line = linear regression line; shaded areas = 95% confidence interval of linear regression line.

Interpretation: there is no association between storage time and PCs, in other words, the storage time does not explain the differences in volatile metabolites.

Figure S4: Influence of duration of mechanical ventilation on principal components derived from GC-MS-1

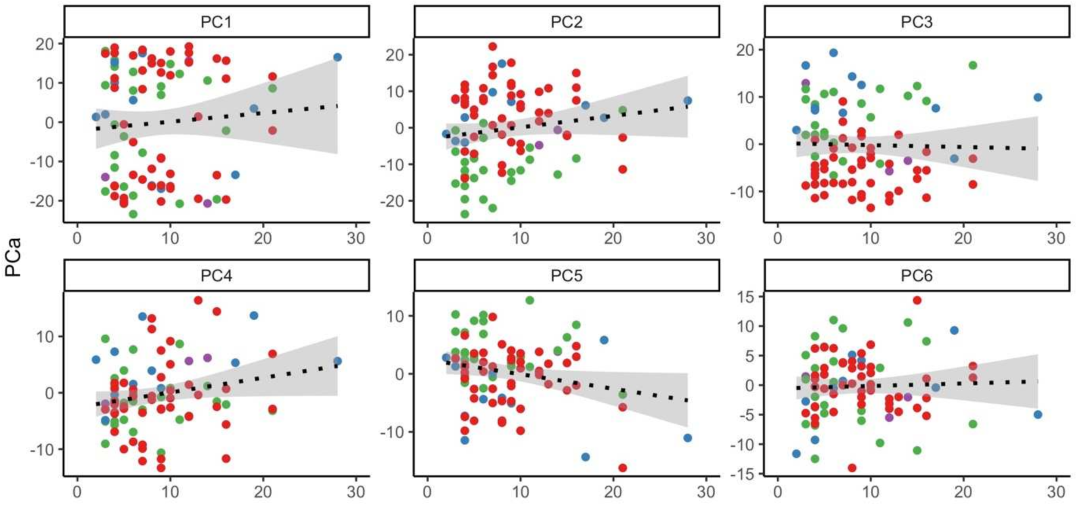

Figure S4: Dots represent the collected breath samples from either AMC Amsterdam (red ●), Manchester Royal Infirmary (MRI) (blue ●), Salford Trust (green ●), and Wythenshawe Hospital (purple ●). Dashed line = linear regression line; shaded areas = 95% confidence interval of linear regression line.

Interpretation: there is no association between duration of mechanical ventilation and PCs, in other words, the duration of ventilation does not explain the differences in volatile metabolites.

van Oort PMP, *et al.* *Thorax* 2021; 77:79–81. doi: 10.1136/thoraxjnl-2021-217362

Figure S5: Influence of GC column change on principal components from GC-MS-1

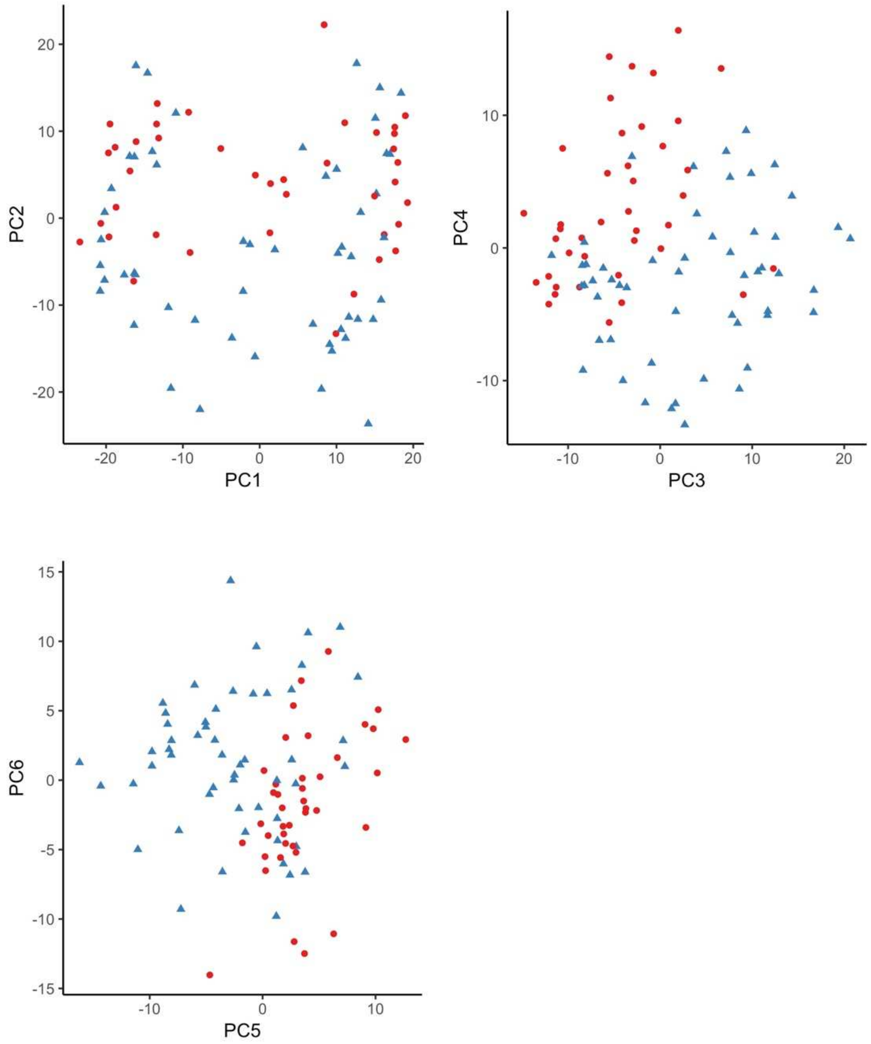

Red dot (●): first column; blue triangle (▲): second column

Interpretation: During the study, the column on GC-MS-1 was changed due to degradation. The change of the column changes the breath profile as indicated by a difference in PC3, PC4, PC5 and PC6. Therefore all subsequent analyses were stratified for each column separately.

van Oort PMP, *et al. Thorax* 2021; 77:79–81. doi: 10.1136/thoraxjnl-2021-217362

Figure S6: Influence of centre on principal components derived from GC-MS-2

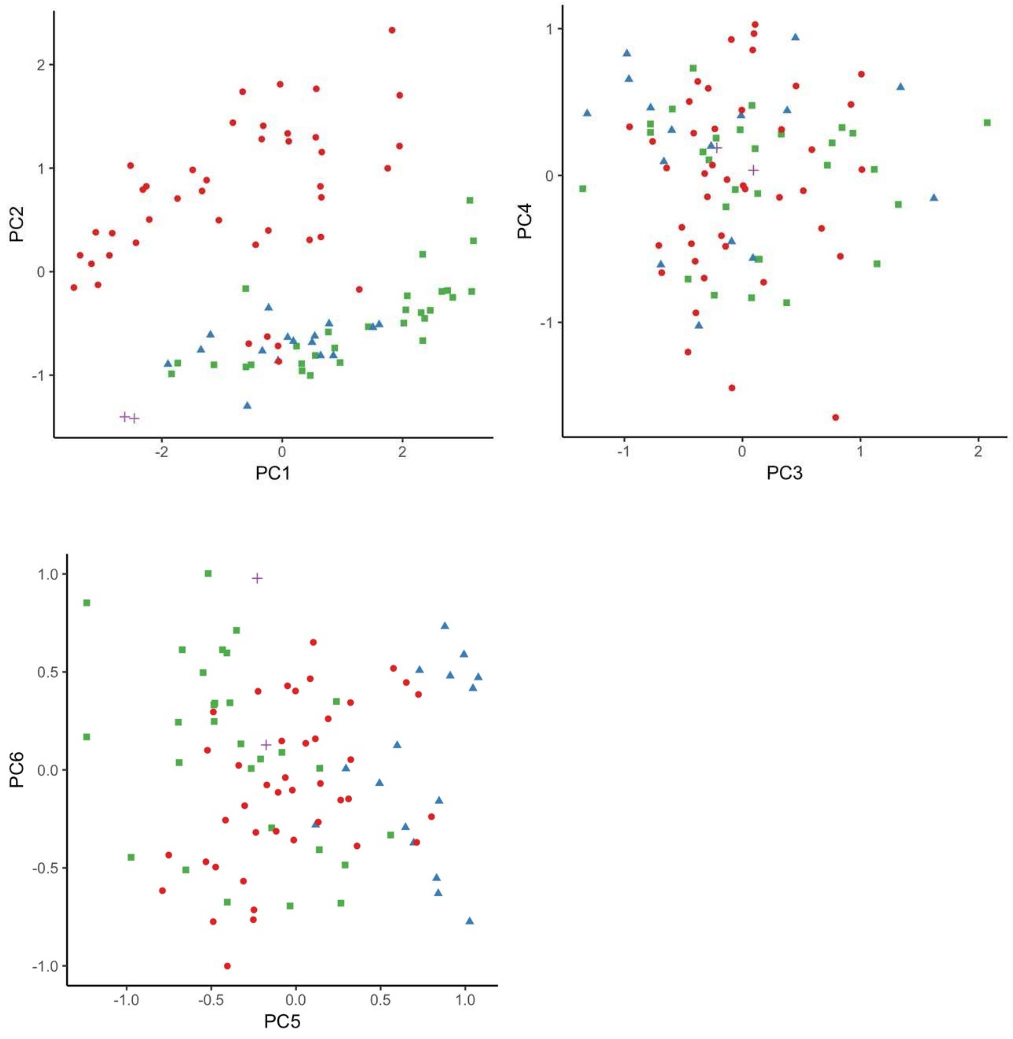

Centres: red dot (●): AMC Amsterdam; blue triangle (▲): Manchester Royal Infirmary; green square (■): Salford; purple cross (+): Wythenshawe hospital

Interpretation: The centres are differentiated based on PC2. Some portion of the variation in exhaled metabolites is thus explained by the centre the patient is recruited.

Figure S7: Influence of analysis date on principal components derived from GC-MS-2

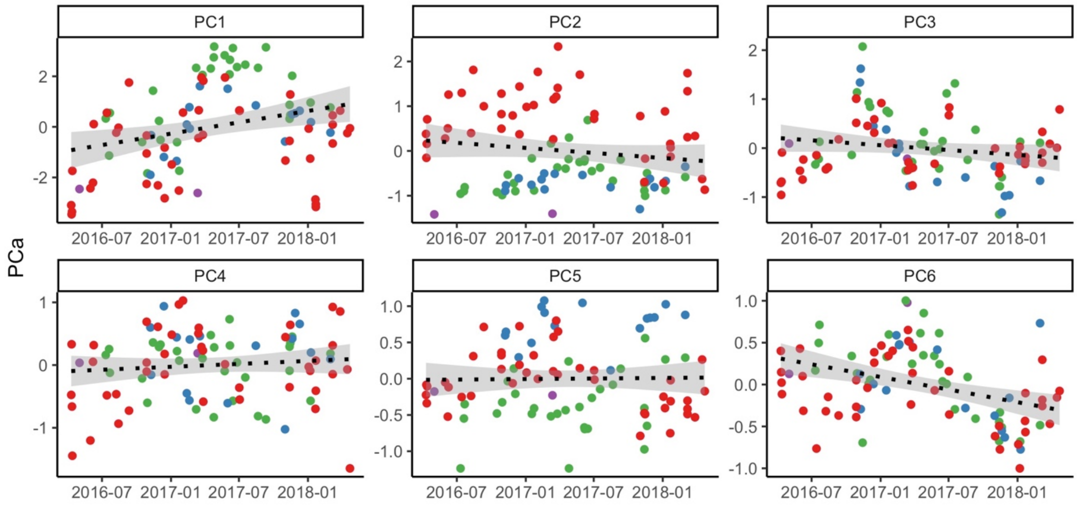

Dots represent the collected breath samples either from AMC Amsterdam (red ●), Manchester Royal Infirmary (MRI) (blue ●), Salford Trust (green ●), and Wythenshawe Hospital (purple ●). Dashed line = linear regression line; shaded areas = 95% confidence interval of linear regression line.

Interpretation: There is no relationship between the time of recruitment and the PCs, so there likely is no systematic and linear change to the detector over time.

van Oort PMP, *et al. Thorax* 2021; 77:79–81. doi: 10.1136/thoraxjnl-2021-217362

Figure S8: Influence of storage time on principal components derived from GC-MS-2

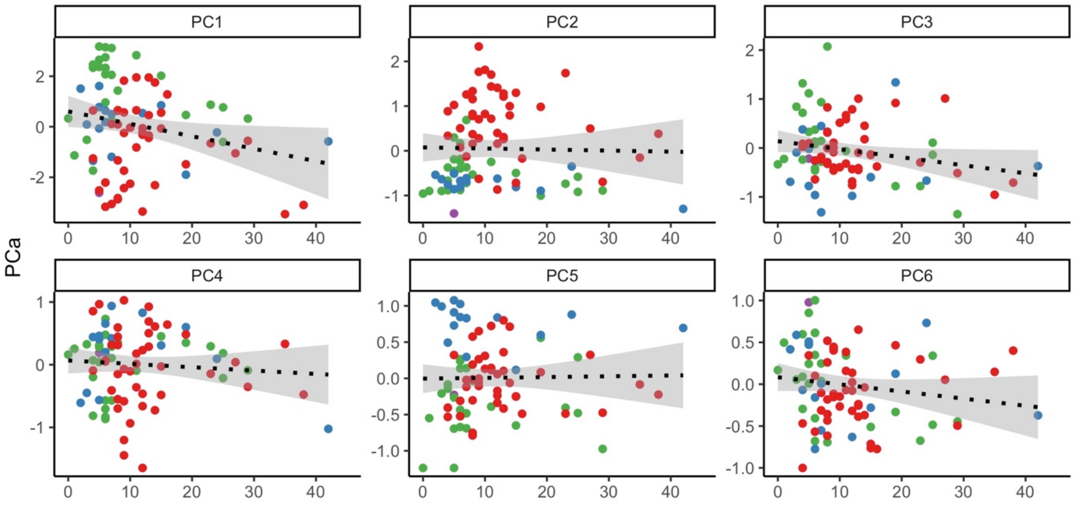

Dots represent the collected breath samples either from AMC Amsterdam (red ●), Manchester Royal Infirmary (MRI) (blue ●), Salford Trust (green ●), and Wythenshawe Hospital (purple ●). Dashed line = linear regression line; shaded areas = 95% confidence interval of linear regression line.

Interpretation: there is no association between storage time and PCs, in other words, the storage time does not explain the differences in volatile metabolites.

Figure S9: Influence of duration of mechanical ventilation on principal components derived from GC-MS-2

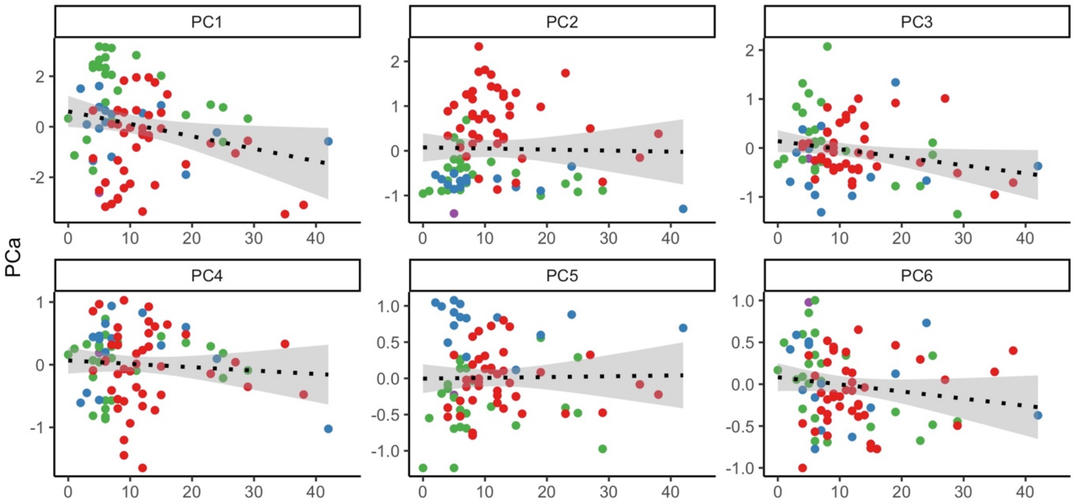

Dots represent the collected breath samples either from AMC Amsterdam (red ●), Manchester Royal Infirmary (MRI) (blue ●), Salford Trust (green ●), and Wythenshawe Hospital (purple ●).
Dashed line = linear regression line; shaded areas = 95% confidence interval of linear regression line.

Interpretation: there is no association between duration of mechanical ventilation and PCs, in other words, the duration of ventilation does not explain the differences in volatile metabolites.

Figure S10: Correct classification rate from SPLS-DA for culture positive vs. culture negative based on 1000 random permutations of the group allocation for GC-MS-1 and -2.

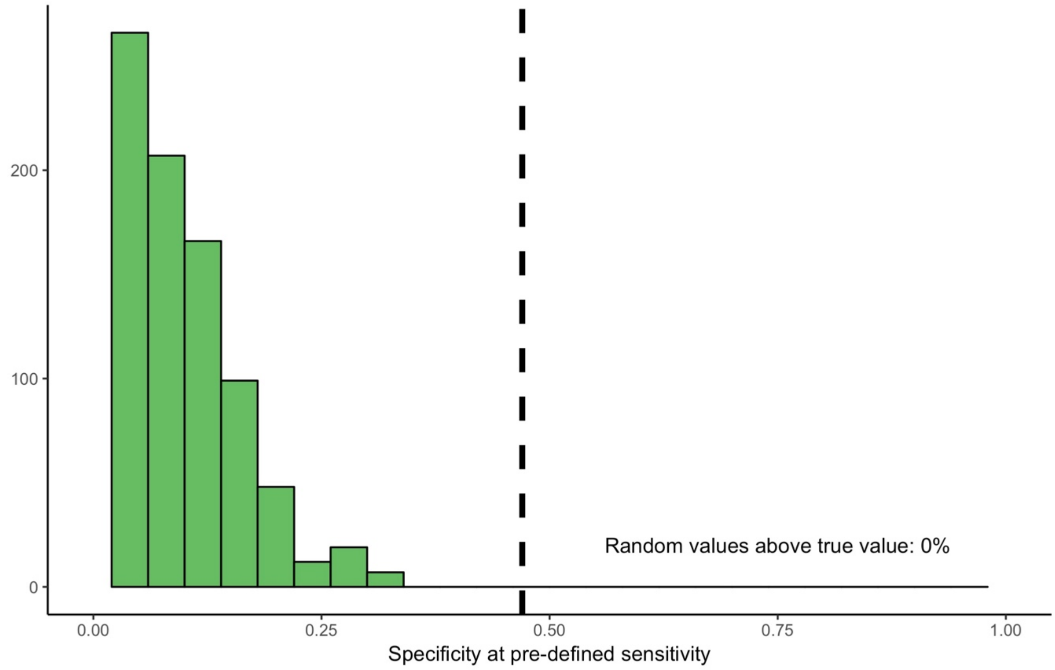

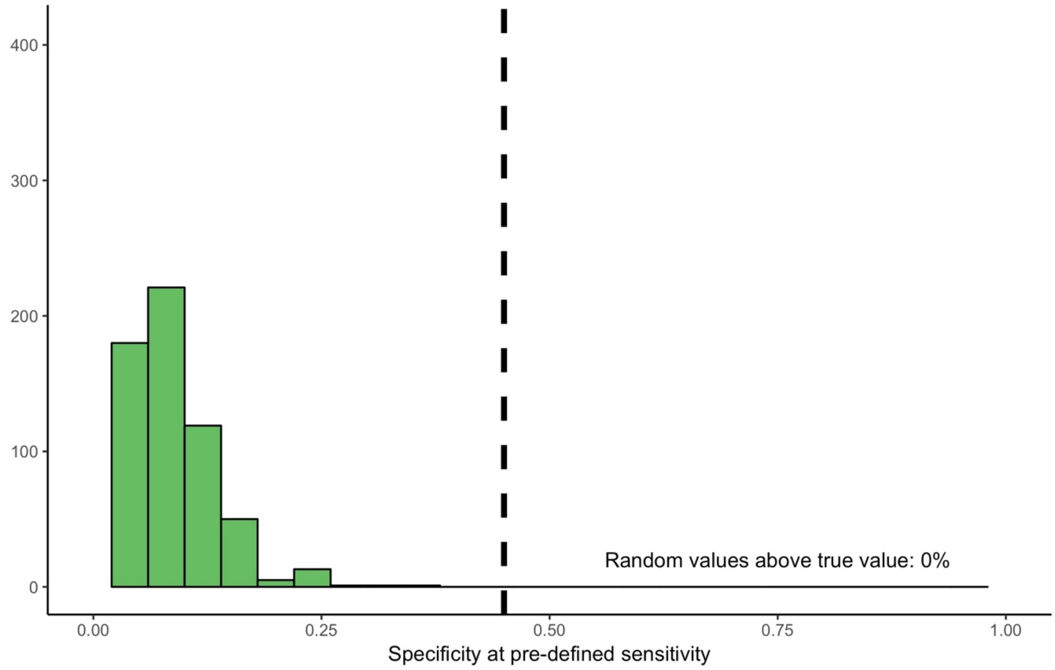

Interpretation: When permutating the labels of confirmed VA-LRTI to a random label there were no scenarios in which similar tech characteristics could be obtained. This suggests that the analysis was not overfit for the data.

van Oort PMP, *et al. Thorax* 2021; 77:79–81. doi: 10.1136/thoraxjnl-2021-217362

Figure S11: ROC curves for culture positive vs. negative.

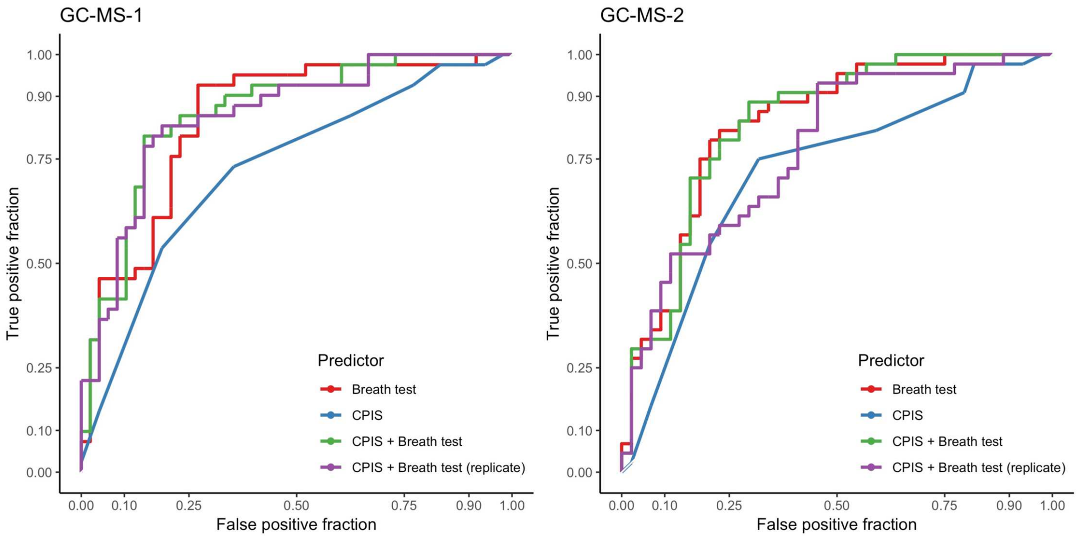

Figure S11. Panel on the left shows the receiver operating characteristics curve for GC-MS-1 and on the right for GC-MS-2. The blue line indicates the performance of the CPIS alone. The red line indicates the breath test. The green line shows the performance for the combination of breath test and CPIS. The purple line is the performance of the same algorithm in a second sample taken from the same patient. For GC-MS-1, the breath test alone delivered an AUROCC 0.86 (95%-confidence interval (CI): 0.79-0.94); CPIS alone AUROCC 0.74 (95%-CI: 0.64-0.84); CPIS + breath test: AUROCC 0.87 (95%-CI: 0.80-0.94); replicate CPIS + breath test: AUROCC 0.85 (95%-CI: 0.77-0.93). For GC-MS-2: CPIS alone: AUROCC 0.72 (95%-CI 0.61-0.83); breath test alone: AUROCC 0.83 (95%-CI: 0.75-0.92); CPIS + breath test AUROCC 0.84 (95%-CI 0.75-0.92); replicate CPIS + breath test: AUROCC 0.77 (95%-CI: 0.67-0.86).

Supplemental references

1. van Oort PMP, Nijsen T, Weda H, et al (2017) BreathDx – molecular analysis of exhaled breath as a diagnostic test for ventilator–associated pneumonia: protocol for a European multicentre observational study. BMC Pulm Med 17:1. https://doi.org/10.1186/s12890-016-0353-7

2. Flahault A, Cadilhac M, Thomas G (2005) Sample size calculation should be performed for design accuracy in diagnostic test studies. J Clin Epidemiol 58:859–862. https://doi.org/10.1016/j.jclinepi.2004.12.009

3. Fowler SJ, Basanta-Sanchez M, Xu Y, et al (2015) Surveillance for lower airway pathogens in mechanically ventilated patients by metabolomic analysis of exhaled breath: a case-control study. Thorax thoraxjnl-2014-206273-. https://doi.org/10.1136/thoraxjnl-2014-206273

4. Du Rand IA, Blaikley J, Booton R, et al (2013) Summary of the British Thoracic Society guideline for diagnostic flexible bronchoscopy in adults. Thorax 68:786–787. https://doi.org/10.1136/thoraxjnl-2013-203629

5. Horváth I, Barnes PJ, Loukides S, et al (2017) A European Respiratory Society technical standard : exhaled biomarkers in lung disease. Eur Respir J 49:. https://doi.org/10.1183/13993003.00965-2016

6. Lindstrom P, Mallard W NIST Chemistry WebBook, NIST Standard Reference Database Number 69. In: 69

7. Sumner LW, Samuel T, Noble R, et al (2007) Proposed minimum reporting standards for chemical analysis Chemical Analysis Working Group (CAWG) Metabolomics Standards Initiative (MSI). Metabolomics 3:211–221. https://doi.org/10.1007/s11306-007-0082-2.Proposed

8. Chun H, Keles S, Keleş S (2010) Sparse partial least squares regression for simultaneous dimension reduction and variable selection. J R Stat Soc Ser B Stat Methodol 72:3–25.

https://doi.org/10.1111/j.1467-9868.2009.00723.x
